# An old retained sharp intrapulmonary foreign body: a case report and literature review

**DOI:** 10.1186/s13019-026-04300-9

**Published:** 2026-06-04

**Authors:** Yiming Lei, Shengteng Shao, Tingshu Jiang, Wei Wang

**Affiliations:** 1 School of clinical medical, Shandong Second Medical University, Weifang, China; 2 Department of Thoracic Surgery, The Affiliated Yantai Yuhuangding Hospital of Qingdao University, No. 20 Yuhuangding East Road, Yantai, 264000 China; 3 Department of Respiratory Medicine, The Affiliated Yantai Yuhuangding Hospital of Qingdao University, Yantai, China

**Keywords:** Intrapulmonary foreign body, Nail, Retained foreign body, Sharp foreign body, Imaging features

## Abstract

**Objective:**

To investigate the clinical characteristics, diagnostic challenges, and management strategies of long-term retained sharp intrapulmonary foreign bodies and to improve clinicians’ understanding of this rare condition.

**Methods:**

We report a case of a 54-year-old male patient presenting with hemoptysis. The diagnosis was established through imaging, multidisciplinary consultation, thoracoscopic surgery, and pathological examination. Additionally, the relevant literature was reviewed to summarize the clinical features of such cases.

**Results:**

Chest CT revealed a dense shadow in the left lower lobe. Following multidisciplinary consultation, a diagnosis of “sharp intrapulmonary foreign body complicated by infection” was made. The patient underwent thoracoscopic left lower lobectomy, during which three nail-like foreign bodies, approximately 1.0–2.0 cm in length, were successfully removed. Postoperative pathology confirmed foreign body-induced pulmonary infection. Further history-taking suggested that the foreign bodies were likely the result of an accidental nail gun injury sustained in a workshop approximately 10 years prior.

**Conclusion:**

Chronic intrapulmonary sharp foreign bodies may remain clinically silent for extended periods, posing diagnostic challenges. A high index of suspicion combined with detailed imaging is essential for diagnosis. Management should be individualized, with surgical intervention considered in symptomatic patients or when complications are likely. For completely asymptomatic patients with a stable foreign body position and no evidence of complications, conservative management with regular imaging follow-up may be a reasonable alternative.

## Introduction

Intrapulmonary foreign bodies are a relatively common clinical emergency, most often caused by aspiration, particularly in children under 3 years of age and in the elderly [1]. Sharp intrapulmonary foreign bodies, however, are introduced into the lung tissue via direct penetration through the chest wall; their formation is therefore “penetrating” rather than “aspirated.” When a sharp foreign body remains in situ for more than one month, it is considered “chronic” or “retained,” and clinical management becomes more complex. Current research, both domestically and internationally, has largely focused on aspirated airway foreign bodies or fresh penetrating foreign bodies. Reports of intrapulmonary foreign bodies possessing the dual characteristics of being both “penetrating” and “chronic” are exceedingly rare in the literature. This article presents a rare case, summarizing its clinical symptoms and imaging features to provide a systematic approach to the diagnosis and treatment of this clinical challenge.

## Case presentation

**Fig. 1:**
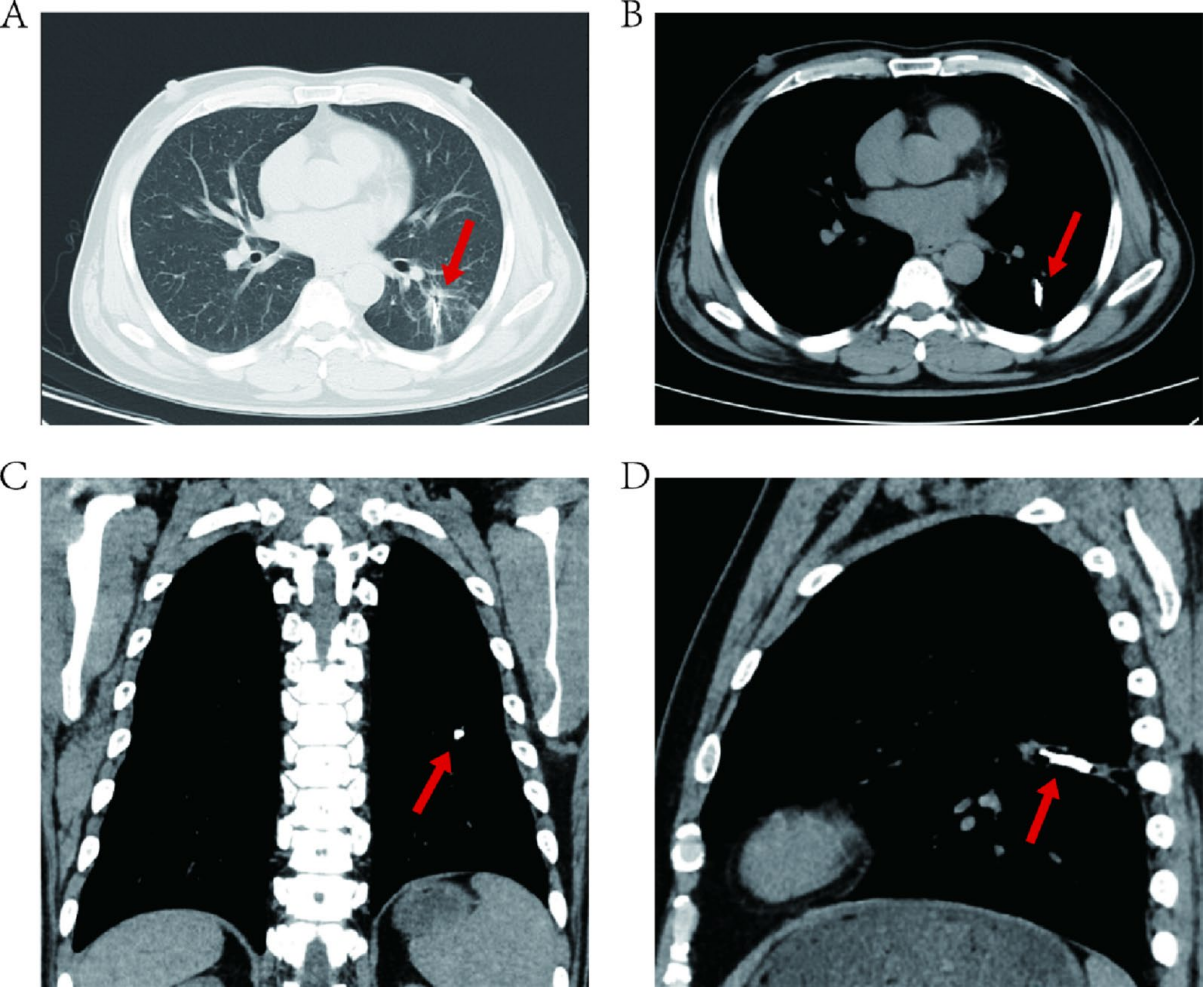
Chest CT images. (**A**) Axial lung window: a linear metallic density is seen in the basal segment of the left lower lobe, surrounded by patchy opacities. (**B**) Axial mediastinal window at the same level confirms the linear high-density foreign body. (**C**) Coronal and (**D**) sagittal reformatted images show the intrapulmonary location of the foreign body

**Fig. 2:**
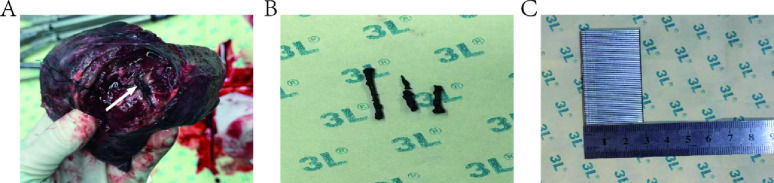
Surgical specimen and retrieved foreign bodies. (**A**) Resected left lower lobe: three metallic nails are visible within the consolidated lung parenchyma. (**B**) The three nails after extraction, measuring 1.0–2.0 cm in length. (**C**) Common carpentry row nails

A 54-year-old male patient was admitted to the Affiliated Yantai Yuhuangding Hospital of Qingdao University, presenting with a chief complaint of “hemoptysis for more than one month.” More than one month prior to admission, the patient experienced two episodes of hemoptysis without any obvious trigger, each consisting of 3–4 expectorations of bright red blood, accompanied by discomfort in the left chest. There were no associated symptoms such as fever, chills, chest tightness, or palpitations. He initially visited another hospital where he received symptomatic treatment including antibiotics, antitussives, expectorants, and hemostatic agents. Several days later, the patient experienced a recurrence of hemoptysis, again producing 3–4 expectorations of bright red blood with left chest discomfort. Further investigation with a chest computed tomography (CT) scan revealed a patchy high-density lesion measuring approximately 2.0 × 1.1 cm in the basal segment of the left lower lobe. The lesion showed spiculated margins with traction on the adjacent pleura, internal vacuole-like lucencies, and a linear dense structure in the distal portion. These findings were suspicious for a foreign body with surrounding inflammatory changes. Left lower lobe pneumonia was also noted (Fig. 1). Three-dimensional reconstruction further confirmed the close anatomical relationship between the foreign body and the surrounding blood vessels and bronchi. The patient had been generally healthy prior to this event with no history of pulmonary disease. The patient had a smoking history of more than 30 years, and pulmonary function tests were normal. There was no past medical history of chronic obstructive pulmonary disease, asthma, or other underlying lung diseases. Physical examination on admission revealed a non-deformed thorax with symmetrical respiratory movements. There was no chest wall tenderness, no visible scar on the chest wall, no enhanced or diminished vocal fremitus, and no sternal tenderness. Cardiopulmonary examination revealed no other significant abnormalities. The patient reported no other complaints. A multidisciplinary team (MDT) was subsequently organized, involving thoracic surgeons, respiratory physicians, and radiologists, to review the imaging collaboratively. The expert panel agreed that the lesion showed characteristic CT features of an intrapulmonary foreign body, including high attenuation (3000–4000 HU), sharp margins, and irregular shape. Furthermore, the chronic encapsulating reaction of the surrounding tissue supported its long-term retention. Based on these findings, a diagnosis of “highly probable sharp intrapulmonary foreign body complicated by infection” was established. The patient was given empirical antibiotic therapy upon admission due to imaging findings suggestive of an intrapulmonary foreign body with secondary infection. Following anti-infective treatment, the patient’s hemoptysis was reduced, but repeat CT revealed no significant absorption of the patchy densities. Given the foreign body’s proximity to pulmonary vessels and its role in causing recurrent hemoptysis, conservative management was deemed ineffective, and surgical intervention was decided upon. The procedure was performed via video-assisted thoracoscopic surgery (VATS). Intraoperatively, partial pleural adhesions were noted, as well as an incomplete interlobar fissure. A consolidated area of lung tissue, approximately 5.0 cm × 4.0 cm, was observed in the basal segment of the left lower lobe, with a slightly retracted overlying visceral pleura. A left lower lobectomy was performed. The main reason for choosing left lower lobectomy over a limited resection (such as wedge resection or anatomical segmentectomy) was that intraoperative exploration revealed the mass was located adjacent to the pulmonary hilum, and long-standing infectious stimulation had caused severe consolidation of the entire basal segment and part of the dorsal segment, making segmental or wedge resection unsafe. Forced limited resection would not only have compromised adequate margins but also risked injuring the adjacent hilar vessels and bronchi. Therefore, to ensure complete removal of the foreign body and the infectious lesion, left lower lobectomy was the safest and most radical choice at that time. Intraoperative localization techniques were not used because the foreign body appeared as a high-density shadow on CT and could be clearly identified by palpation and direct visualization. Upon opening the resected lung specimen extracorporeally, three nail-like foreign bodies, measuring approximately 1.0–2.0 cm in length, were identified (Fig. 2A, B). Postoperative pathological examination of the left lower lobe specimen showed disruption of the alveolar architecture in some areas, with mild atypical hyperplasia of alveolar epithelial cells. Interstitial fibrous tissue and smooth muscle proliferation with hyaline degeneration were observed. Numerous acute and chronic inflammatory cells infiltrated the peribronchiolar and interstitial areas, with focal multinucleated giant cell reactions. These findings were consistent with a foreign body-induced pulmonary infection.

Following the surgical procedure, the nature of the foreign bodies was clarified; they appeared similar to row nails commonly used in carpentry (Fig. 2C). A more detailed medical history was subsequently obtained from the patient. The patient recalled that approximately 10 years prior, he had been working in a furniture factory. One day, after consuming alcohol, he entered a workshop area in violation of safety regulations where nail guns were in operation. A co-worker, noticing he was in a dangerous location within the firing range, escorted him out of the workshop. At the time, he did not experience any immediate discomfort and therefore did not pay particular attention to the incident, remaining unaware that a row nail had penetrated his body. Over the subsequent decade, the patient remained in good health with no specific complaints, until approximately one month prior to this admission when he developed hemoptysis and related symptoms. At the one-year follow-up post-discharge, the patient reported a good recovery with no further discomfort.

## Discussion

Intrapulmonary foreign bodies refer to exogenous objects retained within the respiratory system. Based on their final location, they are primarily classified into two types: airway foreign bodies and foreign bodies within the lung parenchyma. The former are typically caused by aspiration, with the object becoming lodged in the trachea or bronchi. The latter mostly result from penetrating trauma, entering the lung tissue directly through the chest wall [2].

In April 2026, we conducted a literature search in the PubMed database using the following search strategy: (“Foreign Bodies“[MeSH] OR “foreign body“[tiab] OR “foreign bodies“[tiab]) AND (penetrating[tiab] OR penetrating*[tiab]) AND (lung[tiab] OR pulmonary[tiab] OR intrathoracic[tiab] OR “respiratory tract“[tiab]). The inclusion criteria were: (1) case reports or case series; (2) foreign bodies directly entering the lung parenchyma through penetrating trauma; (3) published between January 2016 and April 2026. The exclusion criteria were: (1) foreign bodies entering via the airway or through digestive tract migration; (2) foreign bodies retained only in the chest wall or pleural cavity without entering the lung parenchyma. A total of 35–45 patients meeting the criteria were identified. This analysis focused on cases where foreign bodies directly entered the lung parenchyma through penetrating trauma (e.g., needlestick, gunshot, sharp instrument penetration). The results showed a wide age distribution, with a relatively higher incidence in young to middle‑aged adults (30–50 years). Metallic foreign bodies (bullets/shrapnel, blades, metal fragments) were the most common type; they can remain asymptomatic for long periods and become encapsulated by fibrous tissue. Glass fragments and needles were the second most common, often discovered incidentally. Organic foreign bodies such as fish bones usually migrate via the digestive tract and are often inconspicuous on imaging. Aspirated foreign bodies are typically located in the bronchi and are primarily managed by bronchoscopy. Regarding clinical history, the retention time varied greatly, ranging from hours to 33 years. Most patients presented with obvious symptoms, including hemoptysis, chest pain, or signs of infection. Treatment was almost exclusively surgical removal, with VATS being the mainstream choice due to its minimally invasive advantages. In terms of outcomes, the vast majority of patients recovered well postoperatively, with no severe complications or deaths.

After penetrating the chest wall and entering the lung parenchyma, a penetrating foreign body typically progresses through three stages. The first stage (acute phase): when the foreign body penetrates the chest wall and lung tissue, it causes instantaneous local mechanical injury, which may result in minor bleeding or pneumothorax. However, when the foreign body is small and has low kinetic energy, the elastic recoil of the chest wall and lung tissue allows the puncture tract to close rapidly. Bleeding and air leakage therefore cease spontaneously. Consequently, the patient may be completely asymptomatic. Kakamad et al. reported a case of a bullet retained in the lung parenchyma for 26 years. The patient initially presented only with dull pain at the site of injury, without significant hemoptysis or signs of infection, confirming that in such cases the acute phase can be entirely asymptomatic [3]. Second stage (asymptomatic latent phase): If the foreign body is not removed in time, the body initiates a foreign body reaction. As an inert material, a sharp metallic foreign body within the lung parenchyma induces a chronic mild inflammatory response, activating fibroblasts and promoting collagen deposition. Over months to years, a dense fibrous capsule gradually forms around the foreign body, physically isolating it from the surrounding lung tissue. Otsuka et al. reported a case of an ectopic needle in the lung, demonstrating that foreign bodies can enter the lung parenchyma via transcutaneous penetration and that asymptomatic cases are often discovered incidentally, with a complete fibrous capsule surrounding the foreign body. This fibrous capsule formation explains why patients may remain asymptomatic for long periods [4]. Kivuyo et al. reported a case of a knife blade retained in the thoracic cavity for eight years without any symptoms, further supporting the mechanism by which a fibrous capsule successfully isolates the foreign body from the surrounding tissue [5]. The patient in the present case remained asymptomatic for approximately 10 years, consistent with the above reports. Third stage (symptomatic phase): Over time, the fibrous capsule may gradually become thin and rupture due to long‑term physical stimulation by the foreign body (e.g., friction with respiratory movements) or the accumulation of low‑grade inflammation. Once the integrity of the capsule is disrupted, the sharp edges of the foreign body can directly contact and erode adjacent blood vessels or bronchi, leading to delayed hemoptysis. The surface of the foreign body may also serve as a nidus for bacterial biofilm formation, inducing local suppurative infection when the patient’s immunity is compromised. O’Kane et al. reported a case of a nail gun injury in which a nail was retained in the right hilum adjacent to a branch of the pulmonary artery; the potential erosive risk posed by the sharp edge of the foreign body to the vessel was the main threat, requiring emergency thoracotomy for removal [6]. The patient’s recurrent preoperative hemoptysis suggested rupture of the fibrous capsule, creating a microscopic communication between the foreign body tip and segmental pulmonary vessels. The above mechanism explains why sharp intrapulmonary foreign bodies can suddenly cause symptoms such as hemoptysis or infection after a long asymptomatic period.

The 10‑year asymptomatic retention of the foreign body in this case may be attributed to the following mechanisms: ① a dense fibrous capsule gradually formed around the metallic foreign body, isolating it from the surrounding lung tissue and delaying the occurrence of infection or erosion; ② the nail material was relatively corrosion‑resistant, resulting in a mild local inflammatory response; ③ the foreign body was relatively fixed in position, with minimal frictional irritation from respiratory movements. Risk stratification for complications: Long‑term retention of a sharp metallic foreign body can lead to two major complications – hemorrhage and bronchopleural fistula. The risk of bleeding is closely related to the distance between the tip of the foreign body and the major vessels (pulmonary artery, pulmonary vein). The risk of fistula formation is associated with the degree of infection around the foreign body. In this case, the foreign body was adjacent to the hilar vessels and caused recurrent hemoptysis; therefore, a elective surgical procedure was chosen. Surgical timing: The timing of surgery should be individualized based on the location of the foreign body, the risk of complications, and the patient‘s overall condition. Emergency surgical indications include active massive hemoptysis, penetration of the foreign body into a major vessel, tension pneumothorax, or sepsis. Elective surgery is suitable for asymptomatic or mildly symptomatic patients without active bleeding and with a relatively stable foreign body position. The present patient presented with recurrent minor hemoptysis without emergency signs; after preoperative evaluation, surgery was performed on the fifth day after admission. Role of bronchoscopy: In patients with hemoptysis and a suspected intrapulmonary foreign body, bronchoscopy has dual value: ① to assess whether there is active bleeding in the bronchial tree or whether the foreign body is located in the airway; ② to remove blood clots from the airway and alleviate hemoptysis. In this case, the foreign body was located deep in the lung parenchyma, preoperative imaging showed no protrusion of the foreign body into the bronchus, and there was no active massive hemoptysis; therefore, emergency bronchoscopy was not performed. However, bronchoscopy remains a valuable adjunctive examination when differentiating airway‑source bleeding.

The core characteristic of the foreign body in this case lies in its “penetrating” route, as opposed to the more common “aspirated” pathway. Unlike aspirated foreign bodies, which are often retained in the bronchi, penetrating foreign bodies can be located anywhere within the lung parenchyma, making their clinical presentation more insidious. Studies have shown that 62% of foreign bodies are clearly visible on CT scans [7]. After long-term corrosion, iron nails may become fully encapsulated by fibrous tissue and appear as a nodule or linear opacity on imaging. This appearance can be easily misdiagnosed as an inflammatory pseudotumor, tuberculoma, or even a malignant lung tumor [8–10]. The imaging findings in this case required differentiation from the following conditions: (1) Inflammatory pseudotumor: typically appears as a round or oval soft tissue density with relatively clear borders, but without an extremely high‑density core; (2) Tuberculoma: often located in the apical posterior segment of the upper lobe or the dorsal segment of the lower lobe, with heterogeneous density and possible calcification, but without a metallic foreign body shadow; (3) Lung cancer: usually presents with lobulation and spiculation, and may show pleural indentation, but its density is lower than that of a metallic foreign body. In this case, CT revealed a linear extremely high‑density shadow (CT attenuation of approximately 3000–4000 HU), which, together with the chronic course and recurrent hemoptysis, supported the diagnosis of a foreign body. Unlike inert materials such as plastic, metallic nails pose a persistent and cumulative risk. On one hand, the sharp end may continuously irritate the tissue with respiratory motion, potentially leading to hemorrhage or the formation of a bronchopleural fistula. On the other hand, acting as a permanent nidus of infection, it can trigger severe suppurative infections when the patient’s immunity is compromised [4]. Yao et al. revealed that a longer duration of foreign body retention can increase the risk of heart failure during surgical removal [11]. Delayed treatment leads to long‑term retention of the foreign body in the lung, which in turn causes chronic hypoxia and carbon dioxide retention. Hypoxia can reduce myocardial contractility, while CO₂ retention increases both preload and afterload of the heart, potentially leading to myocardial compensatory hypertrophy and diastolic dysfunction over time. Furthermore, the retained foreign body acts as a persistent source of infection, triggering local recurrent infections (e.g., emphysema, pneumonia). Cytokines and toxins released during the inflammatory response directly damage cardiomyocytes, further weakening cardiac reserve function. When the patient undergoes surgery to remove the foreign body, the anesthetic and surgical stress break the previous compensatory state, leading to aggravated intraoperative hypoxia and a sudden increase in cardiac workload, ultimately inducing acute heart failure. Therefore, for a definitively diagnosed chronic intrapulmonary foreign body, prophylactic surgical removal is recommended provided the patient’s overall condition permits. The successful management of this case also provides a referable practical approach for handling similar situations: comprehensive imaging assessment → MDT risk evaluation → individualized surgical planning → meticulous perioperative management. Similar cases involving an ingested fish bone as an intrapulmonary foreign body have been documented previously. In contrast, metallic foreign bodies like nails are easier to identify on imaging, but they are more destructive and pose a more direct threat to blood vessels. Fish bones tend to induce granuloma formation and recurrent infections [12], whereas nails are more inclined towards “silent” erosion and sudden hemorrhage. This comparison highlights how the “material composition of the foreign body” dictates different considerations in diagnosis and treatment. Furthermore, this case differs from typical traumatic penetrating foreign bodies, which are usually accompanied by a clear history of acute trauma and a wound track. Instances of a chronic intrapulmonary foreign body remaining undetected for over a decade, unbeknownst to the patient, expand our understanding of intrapulmonary foreign bodies.

For benign localized pulmonary lesions, parenchyma‑sparing surgery (such as anatomical segmentectomy or wide wedge resection) is the gold standard in contemporary thoracic surgery. However, in this case, long‑term retention of the foreign body led to chronic infection and local parenchymal consolidation. The extent of the lesion had exceeded a single basal segment and was adjacent to the pulmonary hilum, making limited resection unable to achieve complete removal and adequate safety margins. Intraoperative exploration confirmed dense consolidation of the lung tissue and difficulty in dissecting the intersegmental planes. Therefore, although lobectomy is more invasive, under the specific circumstances of this case it was a necessary choice to ensure radical resection, avoid residual infectious foci, and reduce the risk of recurrence. This decision reflects the principle that the surgical approach must be individualized rather than generalized.

This case is rare and provides valuable insights into clinical diagnosis and management. Chronic intrapulmonary sharp foreign bodies may remain clinically silent for extended periods, posing diagnostic challenges. A high index of suspicion combined with detailed imaging is essential for diagnosis. Management should be individualized, with surgical intervention considered in symptomatic patients or when complications are likely. For completely asymptomatic patients with a stable foreign body position and no evidence of complications, conservative management with regular imaging follow-up may be a reasonable alternative.

## Data Availability

No datasets were generated or analysed during the current study.

## Abbreviations

CT: Computed tomography
MDT: Multidisciplinary team
VATS: Video-assisted thoracoscopic surgery
